# Supplementary material for: Symmetry breaking of the cellular lobes closely relates to phylogenetic structure within green microalgae of the Micrasterias lineage (Zygnematophyceae)
Source: PeerJ. 2018 Dec 7;6:e6098. doi: 10.7717/peerj.6098 (PMC6287601; doi:10.7717/peerj.6098)
Supplement: Table S1 [file peerj-06-6098-s001.doc]

| **Species** | **Code** | **Sampling locality / Strain no.** | **Coordinates of the sampling locality** | **Sampling date** |
| --- | --- | --- | --- | --- |
| *M. americana* | MiAm | a pool in the "Žemlička" quarry, Czech Republic | 48°53'32.26"N, 14°41'54.56"E | 04/2014 |
| *M. apiculata* | MiAp | a pool in the "Langemose" bog, Bornholm, Denmark | 55°07'18.94"N, 14°53'43.71"E | 05/2011 |
| *M. brachyptera* | MiBr | a peat bog pool near Gimån river, Jämtland, Sweden | 62°48'49.29"N, 15°46'08.99"E | 06/2012 |
| *M. crux-melitensis* | MiCM | a pool in the "Březina" fen, Czech Republic | 50°32'54.60"N, 13°54'18.33"E | 06/2011 |
| *M. compereana* | MiCo | a peat bog pool near Hostens, Aquitaine, France | 44°29'37.85"N, 00°37'13.22"W | 03/2009 |
| *M. decemdentata* | MiDc | a pool in the mountainous fen near Gosau, Austria | 47°34'14.00"N, 13°29'45.80"E | 07/2008 |
| *M. denticulata* | MiDe | the "Kohullet" pool, Bornholm, Denmark | 55°07'22.63"N, 14°53'29.67"E | 05/2011 |
| *M. fimbriata* | MiFi | a peat bog pool near Doksy, Czech Republic | 50°34'59.89"N, 14°42'12.89"E | 06/2009 |
| *M. furcata* | MiFu | a peat bog pool near Indal river, Jämtland, Sweden | 63°10'26.69"N, 15°54'29.78"E | 06/2012 |
| *M. jenneri* | MiJe | a peat bog pool near Doksy, Czech Republic | 50°34'33.95"N, 14°40'15.55"E | 06/2009 |
| *M. papillifera* | MiPa | peat bogs near Borkovice, Czech Republic | 49°14'15.32"N, 14°37'54.76"E | 04/2004 |
| *M. radians* var. *bogoriensis* | MiRB | SVCK 389 | -- | isol. 1993 |
| *M. radians* var. *evoluta* | MiRE | SVCK 518 | -- | isol. 2001 |
| *M. rotata* | MiRo | a peat bog pool near Úněšov, Czech Republic | 49°53'50.68"N, 13°10'56.78"E | 05/2013 |
| *M. semiradiata* | MiSm | peat bogs near Borkovice, Czech Republic | 49°14'09.63"N, 14°37'24.19"E | 04/2004 |
| *M. thomasiana* | MiTh | peat bogs near Borkovice, Czech Republic | 49°14'08.72"N, 14°37'24.19"E | 04/2004 |
| *M. truncata* var. *pusilla* | MiTP | NIES 784 | -- | isol. 1988 |
| *M. truncata* var. *quadrata* | MiTQ | a peat bog pool near Doksy, Czech Republic | 50°34'33.95"N, 14°40'15.55"E | 06/2009 |
| *M. truncata* var. *truncata* | MiTT | peat bogs pools near Úněšov, Czech Republic | 49°53'50.68"N, 13°10'56.78"E | 05/2013 |

NIES – Microbial Culture Collection, National Institute for Environmental Studies, Japan (<http://mcc.nies.go.jp/>); SVCK - Culture Collection of Microalgae and Zygnematophyceae, University of Hamburg, Germany (<http://www.mzch-svck.uni-hamburg.de/>)
