# Supplementary material for: Symmetry breaking of the cellular lobes closely relates to phylogenetic structure within green microalgae of the Micrasterias lineage (Zygnematophyceae)
Source: PeerJ. 2018 Dec 7;6:e6098. doi: 10.7717/peerj.6098 (PMC6287601; doi:10.7717/peerj.6098)
Supplement: Table S2 [file peerj-06-6098-s002.doc]

| ***Micrasterias americana*, 7LM, two sublobes** | | | | | | |
| --- | --- | --- | --- | --- | --- | --- |
| **Factor** | **df** | **SS** | **MS** | **R^2^** | **F-ratio** | **p-value** |
| individual | 74 | 0.751 | 0.0102 | 0.747 | 4.486 | 0.001 |
| position | 1 | 0.044 | 0.0443 | 0.044 | 19.589 | 0.001 |
| individual × position | 74 | 0.167 | 0.0023 | 0.167 | 8.038 | 0.001 |
| measurement error | 150 | 0.042 | 0.0003 | 0.042 |  |  |
| ***Micrasterias apiculata*, 7LM, two sublobes** | | | | | | |
| **Factor** | **df** | **SS** | **MS** | **R^2^** | **F-ratio** | **p-value** |
| individual | 74 | 0.292 | 0.0040 | 0.250 | 3.133 | 0.001 |
| position | 1 | 0.745 | 0.7448 | 0.638 | 591.366 | 0.001 |
| individual × position | 74 | 0.093 | 0.0013 | 0.080 | 5.122 | 0.001 |
| measurement error | 150 | 0.037 | 0.0003 | 0.032 |  |  |
| ***Micrasterias apiculata*, 15LM, two sublobes** | | | | | | |
| **Factor** | **df** | **SS** | **MS** | **R^2^** | **F-ratio** | **p-value** |
| individual | 74 | 0.259 | 0.0035 | 0.279 | 2.681 | 0.001 |
| position | 1 | 0.532 | 0.5324 | 0.574 | 407.522 | 0.001 |
| individual × position | 74 | 0.097 | 0.0013 | 0.104 | 5.958 | 0.001 |
| measurement error | 150 | 0.040 | 0.0003 | 0.043 |  |  |
| ***Micrasterias apiculata*, 7LM, lower sublobe** | | | | | | |
| **Factor** | **df** | **SS** | **MS** | **R^2^** | **F-ratio** | **p-value** |
| individual | 74 | 0.425 | 0.0057 | 0.283 | 1.696 | 0.001 |
| position | 1 | 0.705 | 0.7046 | 0.469 | 31.050 | 0.001 |
| individual × position | 74 | 0.250 | 0.0034 | 0.167 | 4.105 | 0.001 |
| measurement error | 150 | 0.122 | 0.0008 | 0.081 |  |  |
| ***Micrasterias apiculata*, 7LM, upper sublobe** | | | | | | |
| **Factor** | **df** | **SS** | **MS** | **R^2^** | **F-ratio** | **p-value** |
| individual | 74 | 0.485 | 0.0066 | 0.546 | 3.241 | 0.001 |
| position | 1 | 0.158 | 0.1578 | 0.178 | 78.064 | 0.001 |
| individual × position | 74 | 0.150 | 0.0020 | 0.169 | 3.190 | 0.001 |
| measurement error | 150 | 0.095 | 0.0006 | 0.107 |  |  |
| ***Micrasterias brachyptera*, 7LM, two sublobes** | | | | | | |
| **Factor** | **df** | **SS** | **MS** | **R^2^** | **F-ratio** | **p-value** |
| individual | 74 | 0.720 | 0.0097 | 0.465 | 1.215 | 0.064 |
| position | 1 | 0.189 | 0.1887 | 0.122 | 23.560 | 0.001 |
| individual × position | 74 | 0.593 | 0.0080 | 0.383 | 26.155 | 0.001 |
| measurement error | 150 | 0.046 | 0.0003 | 0.030 |  |  |
| ***Micrasterias brachyptera*, 15LM, two sublobes** | | | | | | |
| **Factor** | **df** | **SS** | **MS** | **R^2^** | **F-ratio** | **p-value** |
| individual | 74 | 0.679 | 0.0092 | 0.516 | 1.518 | 0.001 |
| position | 1 | 0.131 | 0.1306 | 0.099 | 21.611 | 0.001 |
| individual × position | 74 | 0.447 | 0.0060 | 0.3400 | 15.521 | 0.001 |
| measurement error | 150 | 0.058 | 0.0004 | 0.044 |  |  |
| ***Micrasterias brachyptera*, 7LM, lower sublobe** | | | | | | |
| **Factor** | **df** | **SS** | **MS** | **R^2^** | **F-ratio** | **p-value** |
| individual | 74 | 1.808 | 0.0244 | 0.648 | 2.876 | 0.001 |
| position | 1 | 0.113 | 0.1127 | 0.040 | 13.265 | 0.001 |
| individual × position | 74 | 0.629 | 0.0085 | 0.225 | 5.290 | 0.001 |
| measurement error | 150 | 0.241 | 0.0016 | 0.086 |  |  |
| ***Micrasterias brachyptera*, 7LM, upper sublobe** | | | | | | |
| **Factor** | **df** | **SS** | **MS** | **R^2^** | **F-ratio** | **p-value** |
| individual | 74 | 1.524 | 0.0206 | 0.514 | 2.081 | 0.001 |
| position | 1 | 0.540 | 0.5400 | 0.182 | 54.559 | 0.001 |
| individual × position | 74 | 0.732 | 0.0099 | 0.247 | 8.871 | 0.001 |
| measurement error | 150 | 0.167 | 0.0011 | 0.056 |  |  |
| ***Micrasterias crux-melitensis*, 7LM, two sublobes** | | | | | | |
| **Factor** | **df** | **SS** | **MS** | **R^2^** | **F-ratio** | **p-value** |
| individual | 74 | 0.535 | 0.0072 | 0.694 | 3.261 | 0.001 |
| position | 1 | 0.043 | 0.0430 | 0.056 | 19.365 | 0.001 |
| individual × position | 74 | 0.164 | 0.0022 | 0.213 | 11.536 | 0.001 |
| measurement error | 150 | 0.029 | 0.0002 | 0.037 |  |  |
| ***Micrasterias crux-melitensis*, 15LM, two sublobes** | | | | | | |
| **Factor** | **df** | **SS** | **MS** | **R^2^** | **F-ratio** | **p-value** |
| individual | 74 | 0.479 | 0.0065 | 0.652 | 2.864 | 0.001 |
| position | 1 | 0.055 | 0.0548 | 0.075 | 24.244 | 0.001 |
| individual × position | 74 | 0.167 | 0.0023 | 0.228 | 9.962 | 0.001 |
| measurement error | 150 | 0.034 | 0.0002 | 0.046 |  |  |
| ***Micrasterias crux-melitensis*, 7LM, lower sublobe** | | | | | | |
| **Factor** | **df** | **SS** | **MS** | **R^2^** | **F-ratio** | **p-value** |
| individual | 74 | 0.713 | 0.0096 | 0.224 | 2.123 | 0.001 |
| position | 1 | 2.029 | 2.0288 | 0.637 | 446.830 | 0.001 |
| individual × position | 74 | 0.336 | 0.0045 | 0.106 | 6.428 | 0.001 |
| measurement error | 150 | 0.106 | 0.0007 | 0.033 |  |  |
| ***Micrasterias crux-melitensis*, 7LM, upper sublobe** | | | | | | |
| **Factor** | **df** | **SS** | **MS** | **R^2^** | **F-ratio** | **p-value** |
| individual | 74 | 0.866 | 0.0117 | 0.445 | 2.150 | 0.001 |
| position | 1 | 0.562 | 0.5616 | 0.289 | 103.221 | 0.001 |
| individual × position | 74 | 0.403 | 0.0054 | 0.207 | 7.051 | 0.001 |
| measurement error | 150 | 0.116 | 0.0008 | 0.059 |  |  |
| ***Micrasterias compereana*, 7LM, two sublobes** | | | | | | |
| **Factor** | **df** | **SS** | **MS** | **R^2^** | **F-ratio** | **p-value** |
| individual | 74 | 0.312 | 0.0042 | 0.090 | 1.983 | 0.002 |
| position | 1 | 2.959 | 2.9587 | 0.848 | 1390.048 | 0.001 |
| individual × position | 74 | 0.158 | 0.0021 | 0.045 | 5.242 | 0.001 |
| measurement error | 150 | 0.061 | 0.0004 | 0.017 |  |  |
| ***Micrasterias compereana*, 15LM, two sublobes** | | | | | | |
| **Factor** | **df** | **SS** | **MS** | **R^2^** | **F-ratio** | **p-value** |
| individual | 74 | 0.286 | 0.0039 | 0.117 | 2.015 | 0.001 |
| position | 1 | 1.973 | 1.9726 | 0.806 | 1029.563 | 0.001 |
| individual × position | 74 | 0.142 | 0.0019 | 0.058 | 5.999 | 0.001 |
| measurement error | 150 | 0.048 | 0.0003 | 0.020 |  |  |
| ***Micrasterias compereana*, 7LM, lower sublobe** | | | | | | |
| **Factor** | **df** | **SS** | **MS** | **R^2^** | **F-ratio** | **p-value** |
| individual | 74 | 0.797 | 0.0108 | 0.614 | 3.952 | 0.001 |
| position | 1 | 0.113 | 0.1130 | 0.087 | 41.475 | 0.001 |
| individual × position | 74 | 0.202 | 0.0027 | 0.155 | 2.203 | 0.001 |
| measurement error | 150 | 0.186 | 0.0012 | 0.143 |  |  |
| ***Micrasterias compereana*, 7LM, upper sublobe** | | | | | | |
| **Factor** | **df** | **SS** | **MS** | **R^2^** | **F-ratio** | **p-value** |
| individual | 74 | 0.558 | 0.0075 | 0.617 | 4.196 | 0.001 |
| position | 1 | 0.120 | 0.1199 | 0.132 | 66.662 | 0.001 |
| individual × position | 74 | 0.133 | 0.0018 | 0.147 | 2.870 | 0.001 |
| measurement error | 150 | 0.094 | 0.0006 | 0.104 |  |  |
| ***Micrasterias decemdentata*, 7LM, two sublobes** | | | | | | |
| **Factor** | **df** | **SS** | **MS** | **R^2^** | **F-ratio** | **p-value** |
| individual | 74 | 0.739 | 0.0099 | 0.369 | 2.483 | 0.001 |
| position | 1 | 0.881 | 0.8811 | 0.440 | 218.996 | 0.001 |
| individual × position | 74 | 0.298 | 0.0040 | 0.149 | 7.013 | 0.001 |
| measurement error | 150 | 0.086 | 0.0006 | 0.043 |  |  |
| ***Micrasterias denticulata*, 7LM, two sublobes** | | | | | | |
| **Factor** | **df** | **SS** | **MS** | **R^2^** | **F-ratio** | **p-value** |
| individual | 74 | 0.304 | 0.0041 | 0.286 | 1.940 | 0.001 |
| position | 1 | 0.528 | 0.5277 | 0.497 | 249.026 | 0.001 |
| individual × position | 74 | 0.157 | 0.0021 | 0.148 | 4.308 | 0.001 |
| measurement error | 150 | 0.074 | 0.0005 | 0.069 |  |  |
| ***Micrasterias denticulata*, 15LM, two sublobes** | | | | | | |
| **Factor** | **df** | **SS** | **MS** | **R^2^** | **F-ratio** | **p-value** |
| individual | 74 | 0.260 | 0.0035 | 0.326 | 2.117 | 0.001 |
| position | 1 | 0.355 | 0.3552 | 0.445 | 213.676 | 0.001 |
| individual × position | 74 | 0.123 | 0.0017 | 0.154 | 4.226 | 0.001 |
| measurement error | 150 | 0.059 | 0.0004 | 0.074 |  |  |
| ***Micrasterias denticulata*, 7LM, lower sublobe** | | | | | | |
| **Factor** | **df** | **SS** | **MS** | **R^2^** | **F-ratio** | **p-value** |
| individual | 74 | 0.410 | 0.0055 | 0.273 | 1.793 | 0.001 |
| position | 1 | 0.721 | 0.7206 | 0.480 | 233.525 | 0.001 |
| individual × position | 74 | 0.228 | 0.0031 | 0.152 | 3.208 | 0.001 |
| measurement error | 150 | 0.144 | 0.0010 | 0.096 |  |  |
| ***Micrasterias denticulata*, 7LM, upper sublobe** | | | | | | |
| **Factor** | **df** | **SS** | **MS** | **R^2^** | **F-ratio** | **p-value** |
| individual | 74 | 0.439 | 0.0059 | 0.465 | 5.026 | 0.001 |
| position | 1 | 0.246 | 0.2459 | 0.260 | 208.269 | 0.001 |
| individual × position | 74 | 0.087 | 0.0012 | 0.092 | 1.026 | 0.408 |
| measurement error | 150 | 0.173 | 0.0012 | 0.183 |  |  |
| ***Micrasterias fimbriata*, 7LM, two sublobes** | | | | | | |
| **Factor** | **df** | **SS** | **MS** | **R^2^** | **F-ratio** | **p-value** |
| individual | 74 | 0.412 | 0.0056 | 0.101 | 1.794 | 0.003 |
| position | 1 | 3.406 | 3.4058 | 0.839 | 1097.139 | 0.001 |
| individual × position | 74 | 0.230 | 0.0031 | 0.057 | 32.962 | 0.001 |
| measurement error | 150 | 0.014 | 0.0001 | 0.0035 |  |  |
| ***Micrasterias fimbriata*, 15LM, two sublobes** | | | | | | |
| **Factor** | **df** | **SS** | **MS** | **R^2^** | **F-ratio** | **p-value** |
| individual | 74 | 0.357 | 0.0048 | 0.119 | 1.862 | 0.002 |
| position | 1 | 2.439 | 2.4385 | 0.811 | 941.005 | 0.001 |
| individual × position | 74 | 0.192 | 0.0026 | 0.064 | 19.466 | 0.001 |
| measurement error | 150 | 0.020 | 0.0001 | 0.007 |  |  |
| ***Micrasterias fimbriata*, 7LM, lower sublobe** | | | | | | |
| **Factor** | **df** | **SS** | **MS** | **R^2^** | **F-ratio** | **p-value** |
| individual | 74 | 0.970 | 0.0131 | 0.402 | 2.446 | 0.001 |
| position | 1 | 0.956 | 0.9558 | 0.396 | 178.285 | 0.001 |
| individual × position | 74 | 0.397 | 0.0054 | 0.164 | 8.611 | 0.001 |
| measurement error | 150 | 0.093 | 0.0006 | 0.039 |  |  |
| ***Micrasterias fimbriata*, 7LM, upper sublobe** | | | | | | |
| **Factor** | **df** | **SS** | **MS** | **R^2^** | **F-ratio** | **p-value** |
| individual | 74 | 0.689 | 0.0093 | 0.652 | 6.753 | 0.001 |
| position | 1 | 0.240 | 0.2402 | 0.227 | 174.334 | 0.001 |
| individual × position | 74 | 0.102 | 0.0014 | 0.096 | 7.917 | 0.001 |
| measurement error | 150 | 0.026 | 0.0002 | 0.025 |  |  |
| ***Micrasterias furcata*, 7LM, two sublobes** | | | | | | |
| **Factor** | **df** | **SS** | **MS** | **R^2^** | **F-ratio** | **p-value** |
| individual | 74 | 1.150 | 0.0155 | 0.731 | 3.652 | 0.001 |
| position | 1 | 0.077 | 0.0772 | 0.049 | 18.496 | 0.001 |
| individual × position | 74 | 0.315 | 0.0043 | 0.200 | 20.643 | 0.001 |
| measurement error | 150 | 0.031 | 0.0002 | 0.020 |  |  |
| ***Micrasterias furcata*, 15LM, two sublobes** | | | | | | |
| **Factor** | **df** | **SS** | **MS** | **R^2^** | **F-ratio** | **p-value** |
| individual | 74 | 1.029 | 0.0139 | 0.700 | 3.051 | 0.001 |
| position | 1 | 0.068 | 0.0682 | 0.046 | 14.956 | 0.001 |
| individual × position | 74 | 0.338 | 0.0046 | 0.229 | 19.520 | 0.001 |
| measurement error | 150 | 0.035 | 0.0002 | 0.024 |  |  |
| ***Micrasterias furcata*, 7LM, lower sublobe** | | | | | | |
| **Factor** | **df** | **SS** | **MS** | **R^2^** | **F-ratio** | **p-value** |
| individual | 74 | 1.353 | 0.0183 | 0.774 | 5.056 | 0.001 |
| position | 1 | 0.027 | 0.0266 | 0.015 | 7.353 | 0.002 |
| individual × position | 74 | 0.268 | 0.0036 | 0.153 | 5.347 | 0.001 |
| measurement error | 150 | 0.101 | 0.0007 | 0.058 |  |  |
| ***Micrasterias furcata*, 7LM, upper sublobe** | | | | | | |
| **Factor** | **df** | **SS** | **MS** | **R^2^** | **F-ratio** | **p-value** |
| individual | 74 | 1.564 | 0.0211 | 0.797 | 5.043 | 0.001 |
| position | 1 | 0.007 | 0.0073 | 0.004 | 1.736 | 0.146 |
| individual × position | 74 | 0.310 | 0.0042 | 0.158 | 7.650 | 0.001 |
| measurement error | 150 | 0.082 | 0.0005 | 0.042 |  |  |
| ***Micrasterias jenneri*, 7LM, two sublobes** | | | | | | |
| **Factor** | **df** | **SS** | **MS** | **R^2^** | **F-ratio** | **p-value** |
| individual | 74 | 0.272 | 0.0037 | 0.122 | 1.649 | 0.007 |
| position | 1 | 1.703 | 1.7030 | 0.761 | 762.746 | 0.001 |
| individual × position | 74 | 0.165 | 0.0022 | 0.074 | 3.473 | 0.001 |
| measurement error | 150 | 0.096 | 0.0006 | 0.043 |  |  |
| ***Micrasterias papillifera*, 7LM, two sublobes** | | | | | | |
| **Factor** | **df** | **SS** | **MS** | **R^2^** | **F-ratio** | **p-value** |
| individual | 74 | 0.410 | 0.0055 | 0.345 | 2.878 | 0.001 |
| position | 1 | 0.606 | 0.6064 | 0.511 | 314.917 | 0.001 |
| individual × position | 74 | 0.142 | 0.0019 | 0.120 | 10.161 | 0.001 |
| measurement error | 150 | 0.028 | 0.0002 | 0.024 |  |  |
| ***Micrasterias papillifera*, 15LM, two sublobes** | | | | | | |
| **Factor** | **df** | **SS** | **MS** | **R^2^** | **F-ratio** | **p-value** |
| individual | 74 | 0.409 | 0.0055 | 0.406 | 3.292 | 0.001 |
| position | 1 | 0.445 | 0.4454 | 0.442 | 265.513 | 0.001 |
| individual × position | 74 | 0.124 | 0.0017 | 0.123 | 8.535 | 0.001 |
| measurement error | 150 | 0.029 | 0.0002 | 0.029 |  |  |
| ***Micrasterias papillifera*, 7LM, lower sublobe** | | | | | | |
| **Factor** | **df** | **SS** | **MS** | **R^2^** | **F-ratio** | **p-value** |
| individual | 74 | 0.935 | 0.0126 | 0.733 | 4.817 | 0.001 |
| position | 1 | 0.069 | 0.0688 | 0.054 | 26.226 | 0.001 |
| individual × position | 74 | 0.194 | 0.0026 | 0.152 | 5.041 | 0.001 |
| measurement error | 150 | 0.078 | 0.0005 | 0.061 |  |  |
| ***Micrasterias papillifera*, 7LM, upper sublobe** | | | | | | |
| **Factor** | **df** | **SS** | **MS** | **R^2^** | **F-ratio** | **p-value** |
| individual | 74 | 0.835 | 0.0113 | 0.607 | 6.310 | 0.001 |
| position | 1 | 0.350 | 0.3501 | 0.255 | 195.702 | 0.001 |
| individual × position | 74 | 0.132 | 0.0018 | 0.096 | 4.672 | 0.001 |
| measurement error | 150 | 0.057 | 0.0004 | 0.042 |  |  |
| ***Micrasterias radians* var. *bogoriensis*, 7LM, two sublobes** | | | | | | |
| **Factor** | **df** | **SS** | **MS** | **R^2^** | **F-ratio** | **p-value** |
| individual | 74 | 1.289 | 0.0174 | 0.846 | 6.998 | 0.001 |
| position | 1 | 0.023 | 0.0234 | 0.015 | 9.387 | 0.001 |
| individual × position | 74 | 0.184 | 0.0025 | 0.121 | 13.699 | 0.001 |
| measurement error | 150 | 0.027 | 0.0002 | 0.018 |  |  |
| ***Micrasterias radians* var. *bogoriensis*, 15LM, two sublobes** | | | | | | |
| **Factor** | **df** | **SS** | **MS** | **R^2^** | **F-ratio** | **p-value** |
| individual | 74 | 1.050 | 0.0142 | 0.794 | 5.336 | 0.001 |
| position | 1 | 0.037 | 0.0365 | 0.028 | 13.731 | 0.001 |
| individual × position | 74 | 0.197 | 0.0027 | 0.149 | 10.238 | 0.001 |
| measurement error | 150 | 0.039 | 0.0003 | 0.029 |  |  |
| ***Micrasterias radians* var. *bogoriensis*, 7LM, lower sublobe** | | | | | | |
| **Factor** | **df** | **SS** | **MS** | **R^2^** | **F-ratio** | **p-value** |
| individual | 74 | 1.949 | 0.0263 | 0.740 | 4.425 | 0.001 |
| position | 1 | 0.084 | 0.0844 | 0.032 | 14.178 | 0.001 |
| individual × position | 74 | 0.440 | 0.0060 | 0.167 | 5.574 | 0.001 |
| measurement error | 150 | 0.160 | 0.0011 | 0.061 |  |  |
| ***Micrasterias radians* var. *bogoriensis*, 7LM, upper sublobe** | | | | | | |
| **Factor** | **df** | **SS** | **MS** | **R^2^** | **F-ratio** | **p-value** |
| individual | 74 | 2.413 | 0.0326 | 0.781 | 6.063 | 0.001 |
| position | 1 | 0.188 | 0.1883 | 0.061 | 35.017 | 0.001 |
| individual × position | 74 | 0.398 | 0.0054 | 0.129 | 8.801 | 0.001 |
| measurement error | 150 | 0.092 | 0.0006 | 0.030 |  |  |
| ***Micrasterias radians* var. *evoluta*, 7LM, two sublobes** | | | | | | |
| **Factor** | **df** | **SS** | **MS** | **R^2^** | **F-ratio** | **p-value** |
| individual | 74 | 1.310 | 0.0177 | 0.838 | 6.704 | 0.001 |
| position | 1 | 0.036 | 0.0364 | 0.023 | 13.779 | 0.001 |
| individual × position | 74 | 0.195 | 0.0026 | 0.125 | 18.331 | 0.001 |
| measurement error | 150 | 0.022 | 0.0001 | 0.014 |  |  |
| ***Micrasterias radians* var. *evoluta*, 15LM, two sublobes** | | | | | | |
| **Factor** | **df** | **SS** | **MS** | **R^2^** | **F-ratio** | **p-value** |
| individual | 74 | 1.022 | 0.0138 | 0.798 | 5.069 | 0.001 |
| position | 1 | 0.031 | 0.0312 | 0.024 | 11.445 | 0.001 |
| individual × position | 74 | 0.202 | 0.0027 | 0.157 | 15.399 | 0.001 |
| measurement error | 150 | 0.027 | 0.0002 | 0.021 |  |  |
| ***Micrasterias radians* var. *evoluta*, 7LM, lower sublobe** | | | | | | |
| **Factor** | **df** | **SS** | **MS** | **R^2^** | **F-ratio** | **p-value** |
| individual | 74 | 1.756 | 0.0237 | 0.791 | 5.927 | 0.001 |
| position | 1 | 0.085 | 0.0850 | 0.038 | 21.235 | 0.001 |
| individual × position | 74 | 0.296 | 0.0040 | 0.133 | 7.284 | 0.001 |
| measurement error | 150 | 0.082 | 0.0006 | 0.037 |  |  |
| ***Micrasterias radians* var. *evoluta*, 7LM, upper sublobe** | | | | | | |
| **Factor** | **df** | **SS** | **MS** | **R^2^** | **F-ratio** | **p-value** |
| individual | 74 | 1.769 | 0.0239 | 0.848 | 7.179 | 0.001 |
| position | 1 | 0.003 | 0.0027 | 0.001 | 0.816 | 0.469 |
| individual × position | 74 | 0.246 | 0.0033 | 0.118 | 7.448 | 0.001 |
| measurement error | 150 | 0.067 | 0.0004 | 0.032 |  |  |
| ***Micrasterias rotata*, 7LM, two sublobes** | | | | | | |
| **Factor** | **df** | **SS** | **MS** | **R^2^** | **F-ratio** | **p-value** |
| individual | 74 | 0.415 | 0.0056 | 0.085 | 2.413 | 0.001 |
| position | 1 | 4.303 | 4.3031 | 0.878 | 1852.123 | 0.001 |
| individual × position | 74 | 0.172 | 0.0023 | 0.035 | 28.045 | 0.001 |
| measurement error | 150 | 0.012 | 0.0001 | 0.003 |  |  |
| ***Micrasterias rotata*, 15LM, two sublobes** | | | | | | |
| **Factor** | **df** | **SS** | **MS** | **R^2^** | **F-ratio** | **p-value** |
| individual | 74 | 0.429 | 0.0058 | 0.118 | 2.313 | 0.001 |
| position | 1 | 3.017 | 3.0166 | 0.828 | 1203.659 | 0.001 |
| individual × position | 74 | 0.185 | 0.0025 | 0.051 | 26.459 | 0.001 |
| measurement error | 150 | 0.014 | 0.0001 | 0.004 |  |  |
| ***Micrasterias rotata*, 7LM, lower sublobe** | | | | | | |
| **Factor** | **df** | **SS** | **MS** | **R^2^** | **F-ratio** | **p-value** |
| individual | 74 | 1.153 | 0.0156 | 0.813 | 6.096 | 0.001 |
| position | 1 | 0.022 | 0.0224 | 0.016 | 8.777 | 0.001 |
| individual × position | 74 | 0.189 | 0.0026 | 0.133 | 7.170 | 0.001 |
| measurement error | 150 | 0.053 | 0.0004 | 0.038 |  |  |
| ***Micrasterias rotata*, 7LM, upper sublobe** | | | | | | |
| **Factor** | **df** | **SS** | **MS** | **R^2^** | **F-ratio** | **p-value** |
| individual | 74 | 0.977 | 0.0132 | 0.549 | 9.214 | 0.001 |
| position | 1 | 0.675 | 0.6752 | 0.379 | 471.304 | 0.001 |
| individual × position | 74 | 0.106 | 0.0014 | 0.059 | 9.649 | 0.001 |
| measurement error | 150 | 0.022 | 0.0002 | 0.013 |  |  |
| ***Micrasterias semiradiata*, 7LM, two sublobes** | | | | | | |
| **Factor** | **df** | **SS** | **MS** | **R^2^** | **F-ratio** | **p-value** |
| individual | 74 | 0.587 | 0.0079 | 0.396 | 2.273 | 0.001 |
| position | 1 | 0.600 | 0.6003 | 0.406 | 172.056 | 0.001 |
| individual × position | 74 | 0.258 | 0.0035 | 0.174 | 15.014 | 0.001 |
| measurement error | 150 | 0.035 | 0.0002 | 0.024 |  |  |
| ***Micrasterias semiradiata*, 15LM, two sublobes** | | | | | | |
| **Factor** | **df** | **SS** | **MS** | **R^2^** | **F-ratio** | **p-value** |
| individual | 74 | 0.551 | 0.0074 | 0.456 | 2.430 | 0.001 |
| position | 1 | 0.387 | 0.3872 | 0.321 | 126.431 | 0.001 |
| individual × position | 74 | 0.227 | 0.0031 | 0.188 | 10.591 | 0.001 |
| measurement error | 150 | 0.043 | 0.0003 | 0.036 |  |  |
| ***Micrasterias semiradiata*, 7LM, lower sublobe** | | | | | | |
| **Factor** | **df** | **SS** | **MS** | **R^2^** | **F-ratio** | **p-value** |
| individual | 74 | 0.961 | 0.0130 | 0.466 | 1.681 | 0.001 |
| position | 1 | 0.402 | 0.4017 | 0.195 | 52.006 | 0.001 |
| individual × position | 74 | 0.572 | 0.0078 | 0.277 | 8.923 | 0.001 |
| measurement error | 150 | 0.130 | 0.0009 | 0.063 |  |  |
| ***Micrasterias semiradiata*, 7LM, upper sublobe** | | | | | | |
| **Factor** | **df** | **SS** | **MS** | **R^2^** | **F-ratio** | **p-value** |
| individual | 74 | 1.454 | 0.0196 | 0.522 | 1.689 | 0.001 |
| position | 1 | 0.286 | 0.2863 | 0.103 | 24.607 | 0.001 |
| individual × position | 74 | 0.861 | 0.0116 | 0.309 | 9.529 | 0.001 |
| measurement error | 150 | 0.183 | 0.0012 | 0.066 |  |  |
| ***Micrasterias thomasiana*, 7LM, two sublobes** | | | | | | |
| **Factor** | **df** | **SS** | **MS** | **R^2^** | **F-ratio** | **p-value** |
| individual | 74 | 0.192 | 0.0026 | 0.320 | 1.321 | 0.026 |
| position | 1 | 0.252 | 0.2520 | 0.421 | 128.579 | 0.001 |
| individual × position | 74 | 0.145 | 0.0020 | 0.242 | 27.599 | 0.001 |
| measurement error | 150 | 0.011 | 0.00001 | 0.018 |  |  |
| ***Micrasterias thomasiana*, 15LM, two sublobes** | | | | | | |
| **Factor** | **df** | **SS** | **MS** | **R^2^** | **F-ratio** | **p-value** |
| individual | 74 | 0.172 | 0.0023 | 0.367 | 1.580 | 0.001 |
| position | 1 | 0.176 | 0.1763 | 0.376 | 119.704 | 0.001 |
| individual × position | 74 | 0.109 | 0.0015 | 0.232 | 19.262 | 0.001 |
| measurement error | 150 | 0.011 | 0.0001 | 0.024 |  |  |
| ***Micrasterias thomasiana*, 7LM, lower sublobe** | | | | | | |
| **Factor** | **df** | **SS** | **MS** | **R^2^** | **F-ratio** | **p-value** |
| individual | 74 | 0.388 | 0.0052 | 0.432 | 3.524 | 0.001 |
| position | 1 | 0.373 | 0.3730 | 0.416 | 250.823 | 0.001 |
| individual × position | 74 | 0.110 | 0.0015 | 0.123 | 8.597 | 0.001 |
| measurement error | 150 | 0.026 | 0.0002 | 0.029 |  |  |
| ***Micrasterias thomasiana*, 7LM, upper sublobe** | | | | | | |
| **Factor** | **df** | **SS** | **MS** | **R^2^** | **F-ratio** | **p-value** |
| individual | 74 | 0.352 | 0.0048 | 0.731 | 4.465 | 0.001 |
| position | 1 | 0.025 | 0.0253 | 0.052 | 23.721 | 0.001 |
| individual × position | 74 | 0.079 | 0.0011 | 0.164 | 6.229 | 0.001 |
| measurement error | 150 | 0.026 | 0.0002 | 0.053 |  |  |
| ***Micrasterias truncata* var. *pusilla*, 7LM, two sublobes** | | | | | | |
| **Factor** | **df** | **SS** | **MS** | **R^2^** | **F-ratio** | **p-value** |
| individual | 74 | 1.412 | 0.0191 | 0.399 | 3.007 | 0.001 |
| position | 1 | 1.582 | 1.5820 | 0.448 | 249.271 | 0.001 |
| individual × position | 74 | 0.470 | 0.0064 | 0.133 | 13.506 | 0.001 |
| measurement error | 150 | 0.070 | 0.0005 | 0.020 |  |  |
| ***Micrasterias truncata* var. *quadrata*, 7LM, two sublobes** | | | | | | |
| **Factor** | **df** | **SS** | **MS** | **R^2^** | **F-ratio** | **p-value** |
| individual | 74 | 0.275 | 0.0037 | 0.441 | 1.703 | 0.001 |
| position | 1 | 0.140 | 0.1400 | 0.224 | 64.109 | 0.001 |
| individual × position | 74 | 0.162 | 0.0022 | 0.259 | 6.849 | 0.001 |
| measurement error | 150 | 0.048 | 0.0003 | 0.077 |  |  |
| ***Micrasterias truncata* var. *truncata*, 7LM, two sublobes** | | | | | | |
| **Factor** | **df** | **SS** | **MS** | **R^2^** | **F-ratio** | **p-value** |
| individual | 74 | 0.382 | 0.0052 | 0.476 | 1.979 | 0.001 |
| position | 1 | 0.182 | 0.1824 | 0.228 | 70.004 | 0.001 |
| individual × position | 74 | 0.193 | 0.0026 | 0.241 | 8.722 | 0.001 |
| measurement error | 150 | 0.045 | 0.0003 | 0.056 |  |  |
| ***Micrasterias truncata* var. *truncata*, 15LM, two sublobes** | | | | | | |
| **Factor** | **df** | **SS** | **MS** | **R^2^** | **F-ratio** | **p-value** |
| individual | 74 | 0.437 | 0.0059 | 0.549 | 2.455 | 0.001 |
| position | 1 | 0.125 | 0.1250 | 0.157 | 52.014 | 0.001 |
| individual × position | 74 | 0.178 | 0.0024 | 0.223 | 6.394 | 0.001 |
| measurement error | 150 | 0.056 | 0.0004 | 0.071 |  |  |
| ***Micrasterias truncata* var. *truncata*, 7LM, lower sublobe** | | | | | | |
| **Factor** | **df** | **SS** | **MS** | **R^2^** | **F-ratio** | **p-value** |
| individual | 74 | 0.806 | 0.0109 | 0.424 | 2.130 | 0.001 |
| position | 1 | 0.562 | 0.5617 | 0.295 | 109.926 | 0.001 |
| individual × position | 74 | 0.378 | 0.0051 | 0.199 | 4.925 | 0.001 |
| measurement error | 150 | 0.156 | 0.0010 | 0.082 |  |  |
| ***Micrasterias truncata* var. *truncata*, 7LM, upper sublobe** | | | | | | |
| **Factor** | **df** | **SS** | **MS** | **R^2^** | **F-ratio** | **p-value** |
| individual | 74 | 1.095 | 0.0148 | 0.613 | 2.378 | 0.001 |
| position | 1 | 0.070 | 0.0698 | 0.039 | 11.215 | 0.001 |
| individual × position | 74 | 0.461 | 0.0062 | 0.258 | 5.846 | 0.001 |
| measurement error | 150 | 0.160 | 0.0011 | 0.089 |  |  |

df degrees of freedom, SS sum of squares, MS mean squares, R^2^ percentage of variance explained by the effect, F pseudo-F ratio, p-value percentile of the effect size in the random distribution of F values
