# Supplementary figures and images for: Symmetry breaking of the cellular lobes closely relates to phylogenetic structure within green microalgae of the Micrasterias lineage (Zygnematophyceae)
Source: PeerJ. 2018 Dec 7;6:e6098. doi: 10.7717/peerj.6098 (PMC6287601; doi:10.7717/peerj.6098)

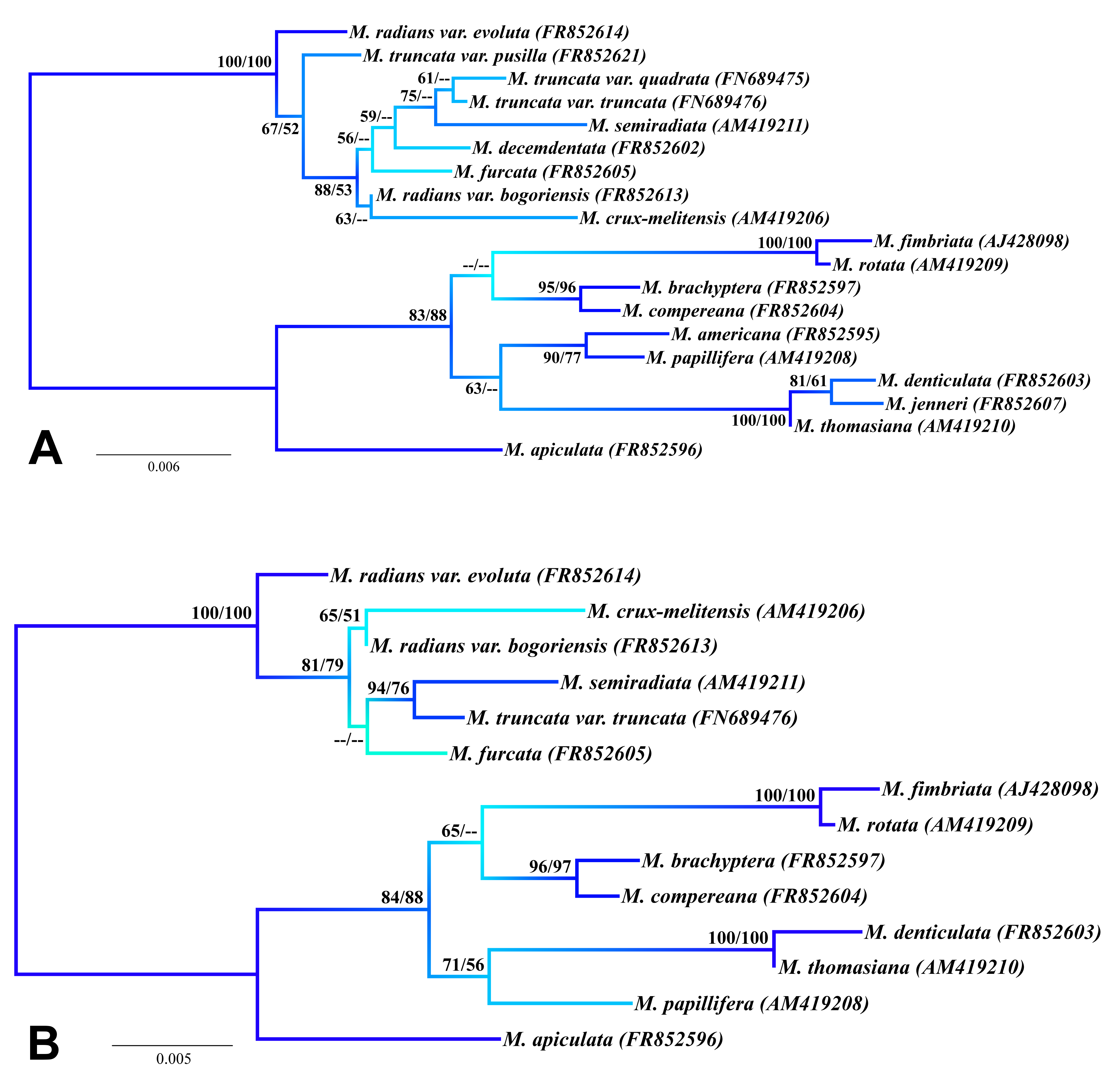

Supplement: Figure S1 — (A) The analysis of 19 taxa with cells differentiated at least into the 2nd-order lobules. (B) The analysis of 14 taxa with cells differentiated at least into the 3rd-order lobules. The bootstrap values of individual nodes correspond to maximum likelihood and maximum parsimony analyses. Blue shading corresponds to ML bootstrap values of the nodes. [file peerj-06-6098-s003.png]
